# All for One: Contributions of Age, Socioeconomic Factors, Executive Functioning, and Social Cognition to Moral Reasoning in Childhood

**DOI:** 10.3389/fpsyg.2016.00227

**Published:** 2016-03-08

**Authors:** Evelyn Vera-Estay, Anne G. Seni, Caroline Champagne, Miriam H. Beauchamp

**Affiliations:** ^1^Department of Psychology, University of MontrealMontreal, QC, Canada; ^2^Sainte-Justine Hospital Research Center, MontrealQC, Canada

**Keywords:** moral reasoning, executive functions, affect recognition, theory of mind, childhood

## Abstract

Moral reasoning (MR) is a socio-cognitive skill essential to appropriate social functioning in childhood, and evolves in quality and complexity during ontogenetic development. Past research suggests that MR is related to age, socioeconomic factors, as well as some social and cognitive skills, such as executive functioning (EF), theory of mind (ToM), empathy, and affect recognition. However, their contributions have been studied in silos rather than comprehensively, with little integration of the relative and combined contribution of these skills to MR. Furthermore, few studies have addressed the putative links between these factors in childhood, a period during which these skills are in maturation. The aim of this study was to explore what factors predict moral maturity in typically developing children (*n* = 76, 47.4% males, *M* = 9.2, *SD* = 1.67 years), explore the potential moderating and mediating role of executive functions and social cognition in the relationship between age and MR maturity, and identify the specific contributions of age, socioeconomic factors, EF, and social cognition, using an innovative visual MR assessment tool (So-Moral). The results indicate that MR maturity was correlated with age, EF (inhibition, verbal fluency, and attentional control), and social cognition (ToM and affect recognition). Neither EF nor social cognition moderated the effect of age on MR maturity. However, verbal fluency and third-order false beliefs had a moderating role in this link. MR maturity in children was predicted by three variables from each of the three domains: age, verbal fluency, and third-order ToM. These results contribute to a better understanding of the underpinnings of MR during childhood, suggesting that MR is not reducible to general developmental factors such as age, but that higher order skills, such EF and social cognition also contribute to moral maturity. The findings have relevance for both typically developing and clinical populations in which social skills may be reduced, as well as for the identification of potential loci for intervention in children at-risk for socially maladaptive behaviors.

## Introduction

Humans, as social beings and moral agents, are faced with making their first moral decisions much before attaining cognitive, emotional, and social maturity. In everyday life, children have to evaluate morally relevant situations in order to make decisions concerning social behaviors such as fair play, admit their wrongdoings, participate or not in bullying, and so on, by using abilities that are not yet consolidated. Current research demonstrates that children are able to differentiate moral issues from social conventions and personal choices (Turiel, 2002), and that their moral understanding increases during ontogenetic development, presumably growing in parallel with cognitive abilities and social cognition (Lane et al., 2010). In addition, some studies suggest that demographic factors such as maternal education and social risk could influence moral understanding (Dunn et al., 2000; Arsenio and Gold, 2006; Hinnant et al., 2013).

Thus, moral reasoning (MR) in childhood is likely to be underpinned by cognitive, socio-cognitive, and affective capacities in maturation, as well as by demographic factors. However, whereas past research suggests an association between MR skills and the development of some social and cognitive skills, such as executive functioning (EF; Hinnant et al., 2013), theory of mind (ToM; Baird and Astington, 2004), empathy (Hoffman, 2000), and affect recognition (Blair et al., 2001a), their contributions have been studied in silos rather than comprehensively, with little integration of the relative and combined contribution of these skills to MR, or the potential mediation and moderation effects of these skills in the relationship between age and MR skills. Understanding the building blocks of MR skills in childhood has relevance for both typically developing and clinical populations in which social skills may be reduced.

### Moral Reasoning and Socioeconomic Factors

Broadly, some studies indicate that moral understanding may be affected by demographic factors, such as socioeconomic status and social risk in particular (Haidt et al., 1993; Dunn et al., 2000; Arsenio and Gold, 2006). However, concerning MR skills specifically, some studies have found a positive link with socioeconomic status (Cui et al., 2015) and maternal education (Hinnant et al., 2013), while others have not shown such an association (Caravita et al., 2012; Malti et al., 2013; Vera-Estay et al., 2014), including a large-scale longitudinal study from childhood to early adolescence (Daniel et al., 2014).

### Moral Reasoning and Socio-Cognitive Skills

Social cognition is a broad construct referred to as the human capacity to think about others’ thoughts, intentions, feelings, attitudes, and perspectives, enabling us to interact in the social world (Sharp et al., 2008). MR is a specific component of social cognition, defined as the thinking mechanism through which moral judgments about right and wrong are attained (Moll et al., 2005). Elementary manifestations of MR can be observed as early as infancy (Malti and Latzko, 2010; Decety et al., 2012), but it is during early childhood that MR skills begin to be more explicit, as children interact verbally with peers, siblings and adults, resolve conflicts and negotiate social situations, leading them to experience concepts such as reciprocity, fairness, and justice (Killen and Rutland, 2011). MR skills continue to increase during middle childhood, supporting prosocial tendencies toward peers and adults (Miller et al., 1996). According to Social Domain theory (Turiel, 2002), during this period, children learn to distinguish and progressively coordinate three main forms of social knowledge: moral, social, and psychological, adding flexibility and nuance to their interpretation of the social world. Moral knowledge related to fairness, justice, welfare and rights, changes during childhood. Thus, whereas younger children tend to evaluate moral situations based on concrete concepts of harm and welfare, older children favor abstract concepts of justice and rights (Turiel, 2002; Killen and Rutland, 2011). This evolution is likely influenced by global cognitive development, in particular by the evolution of high order cognitive skills such as executive functions (EF), a broad range of skills that are implicated in regulating thinking and behavior (Kerr and Zelazo, 2004), and by other socio-cognitive functions developing in parallel.

In addition to MR, socio-cognitive skills include several other mental processes used to perceive and process social cues, stimuli, and environments (Beauchamp and Anderson, 2010). Research in developmental psychopathology has shown that poor social functioning may be in part explained by deficits in these functions (for a review, see Sharp et al., 2008). Considering that even normal variations in socio-cognitive skills could potentially translate into inter-individual differences in social functioning, the current study focused on the associations between MR skills during childhood and three fundamental aspects of social cognition: affect recognition, empathy, and ToM.

#### Affect Recognition

Humans are born with an innate preference for faces (Johnson and Morton, 1991). They communicate important information about the emotional state of individuals, which is essential for accurately interpreting social situations. Facial affect recognition develops rapidly during infancy and early childhood (Herba et al., 2006) and continues to evolve during late childhood, adolescence, and even adulthood (Thomas et al., 2007). The evolution of this skill appears to be influenced by other neurocognitive abilities, specifically by EF, as demonstrated in a study of 1000 healthy individuals (6 to 91 years; Mathersul et al., 2009). Consistent with these findings, Rosenqvist et al. (2014) found that EF, language, and ToM skills are significant predictors of children’s affect recognition.

Recognizing others’ emotions in moral conflicts is likely to influence MR. Deficits in facial affect recognition are associated with psychopathy in youth and adult offenders (Hastings et al., 2008; Bowen et al., 2014), but also in children with psychopathic tendencies, who appear to be less sensitive to sad and fearful facial expressions compared to control groups (Blair et al., 2001a; Stevens et al., 2001), suggesting a link between affect recognition and morality.

#### Empathy

“Empathy is the ability to experience and understand what others feel without confusion between oneself and others” (Decety and Lamm, 2006, p. 1146). Empathy is critical to regulating behavior during social interactions and has been related to prosocial MR, moral motivation and prosocial behavior (Eisenberg and Mussen, 1978; Eisenberg and Fabes, 1990; Miller et al., 1996; Roberts and Strayer, 1996; Hoffman, 2000; Eisenberg et al., 2002; Eisenberg, 2003; Beauchamp et al., 2013). However, research demonstrates a complex relation between empathy and morality. Globally, empathy contributes to MR because it informs on the emotional state of the actors involved in social situations. These emotional states include victim’s suffering, as well as victimizer emotional experience toward the moral conflict, which may prevent the rationalization of moral transgressions. Despite this, empathic tendencies could also interfere with MR, introducing in-group bias and justifying immoral behaviors (Decety and Cowell, 2015). Moreover, given that the general term “empathy” is used for describing several more specific affective and cognitive concepts, the relationship between empathy and other cognitive and behavior variables tends to be blurred (Decety and Cowell, 2014).

#### Theory of Mind

Theory of mind refers to the cognitive ability that allows us to understand other people as intentional, perceptive, and emotional agents, or to interpret their minds in terms of intentional, perceptual, or feeling states (Moll et al., 2005). ToM skills involve progressive abilities that enable inference of other’s mental states at different levels of recursive thinking (*first-order belief*, John thinks X; *second-order belief*, John thinks Mary thinks X; *third-order belief*, John thinks what Mary thinks that he thinks X, and so on), helping to understand the complexity of human social interactions. However, when we infer others’ mental states, we must also determine whether they differ from reality or from our own knowledge about the world, an ability called “false belief understanding.” First-, second-, and third-order false belief tasks assess false belief understanding at different levels of ToM (Korkmaz, 2011).

Whereas first order ToM is generally mastered by healthy children at around 5 years of age, second and third-order ToM, as well as false belief understanding, continue to develop through childhood and adolescence (Liddle and Nattle, 2006; Dumontheil et al., 2010), supported by the development of social experience and specific high-level cognitive abilities such as EF. Thus, according to a meta-analysis conducted by Devine and Hughes (2014), EF in the preschool years is a significant predictor of false belief understanding at school-age, and these findings are consistent across different EF tasks and cultures. Theoretically, cognitive inhibition is a central mechanism in false belief understanding, because it requires inhibition of one’s own knowledge in order to think about other’s mental states (Birch and Bloom, 2004). ToM and EF are considered to be the cognitive bases of social perspective-taking (Gibbs, 2013).

There is growing evidence of the association between MR and ToM skills (Lagattuta, 2005; Wainryb and Brehl, 2006; Killen et al., 2011; Smetana et al., 2012). ToM is potentially implicated in the evaluation of moral situations, because it provides information about the intention and motivation of others’, two key aspects in the evaluation of moral values (Baird and Astington, 2004). A cognitive neuroscience study (Young et al., 2007) confirms this view suggesting that moral judgment depends on cognitive processes mediated by brain areas related to belief attribution and ToM. Research in typically developing children indicates that as children grow, they increasingly incorporate information about mental states in their moral evaluations (Cushman et al., 2013). In addition, children who rely on more advanced ToM, including false belief, have better moral understanding (Dunn et al., 2000) and more mature MR (Baird and Astington, 2004), which is consistent with the results of a longitudinal study conducted by Lane et al. (2010) in which ToM skills predicted more mature (societally-oriented) MR in children.

### Moral Reasoning and Executive Functions

As mentioned above, the intense evolution of EF, and in particular, inhibition, during infancy and early childhood is associated with the development of socio-cognitive skills (Barkley, 1997; Beauchamp and Anderson, 2010), with better EF positively associated with social competence (Kochanska et al., 2000), ToM (Carlson and Moses, 2001; Devine and Hughes, 2014), and moral understanding (Decety and Howard, 2013). Moreover, EF has been shown to be a significant predictor of later social functioning, contributing to social adjustment and resilience (Eisenberg et al., 2004; Martel et al., 2007; Miller and Hinshaw, 2010). However, there are few studies addressing the specific link between EF and MR in childhood and their results support the need for further investigation. A recent study in healthy school-aged children demonstrated that executive skills are positively associated with MR (Hinnant et al., 2013), consistent with findings in school-aged children with developmental disabilities and frontal lesions (Couper et al., 2002; Kretschmer et al., 2014). Recent research also supports this link in adolescents, in whom more mature MR was found to be associated with several executive functions (conceptual reasoning, cognitive flexibility, verbal fluency, and feedback utilization), with flexibility and verbal fluency independently predicting MR maturity, after controlling for the effects of age and intelligence (Vera-Estay et al., 2014). However, these associations have not been tested in children, nor do we know how they play out when socio-cognitive skills are considered in combination.

## The Current Study

The present study contributes to developmental socio-cognitive research by exploring the combined and independent contributions of age and socioeconomic status, cognitive and socio-cognitive variables to MR maturity skills using a novel, developmentally appropriate, visual task. Specifically, the study aimed to: (1) evaluate the association between MR, age, socioeconomic variables (family income and parental education), EF (attentional control, inhibition, and verbal fluency), and social cognition (empathy, ToM, and affect recognition); (2) evaluate whether EF moderates or mediates the relationship between age and MR skills (3) evaluate whether executive and social cognition variables mediate or moderate the relationship between age and MR skills (4) assess the contribution of social cognition to MR maturity, after controlling for EF and age. In line with current literature on MR development described above, it was expected that age, executive and socio-cognitive functions would be positively correlated with MR maturity. Considering previous results using the So-Moral task (Vera-Estay et al., 2014), we expected that socioeconomic variables would not be associated with MR scores. Regarding moderation and mediation effects, we expected that EF and social cognition variables would play a mediating and moderating role in the relationship between age and MR skills. We also expected that age and EF would predict MR maturity and specifically, that verbal fluency would have an independent contribution to MR scores. Concerning social cognition, on the basis of current literature (Dunn et al., 2000; Baird and Astington, 2004; Lane et al., 2010; Cushman et al., 2013) we expected that it would predict MR scores, with ToM making the largest contribution.

## Materials and Methods

### Participants

Seventy-six participants between 6 and 12 years (47.4% males, *M* = 9.2, *SD* = 1.67 years) participated in this study. All participants and their families were French speakers. They were predominantly Caucasian (95%), had no history of any psychiatric or neurological condition, had IQ levels in the low to high average range (87–129, *M* = 108.6, *SD* = 9.9) and were primarily from middle-class families according to their income (Statistics Canada, 2015). Participants were identified and recruited through regular primary schools in Quebec, Canada. All parents provided written informed consent prior to participation. Participants were compensated $30 for their participation. The Research Ethics Committee of the Faculty of Arts and Science at the University of Montreal approved the study.

### Measures

To control for order effects in the assessments, two counterbalancing procedures were applied randomly across participants: one to the order of the whole testing session, and one to the order of the dilemmas in the So-Moral task (see below). The following measures were administered.

#### Demographic and Developmental Questionnaire

Parents’ of participants completed a questionnaire pertaining to their child’s medical, developmental, and social history, as well parents’ education level, ethnicity, and income.

#### Intellectual Functioning

The Wechsler Abbreviated Scale of Intelligence (WASI, Wechsler, 1999) was used to provide an estimate of general intellectual ability based on the Vocabulary and Matrix Reasoning subtests (IQ, *M* = 100, *SD* = 15).

#### Theory of Mind

To assess children’s ability to infer other people’s mental states, the Theory of Mind Picture Stories task (Brune, 2005; Bechi et al., 2012) was administered. This task consists of six cartoon picture storied depicted on four cards each. Two are stories in which two characters cooperate, two are stories in which one character deceives another, and two stories show two characters cooperating to deceive a third. The cards are presented face-down in mixed order; participants are asked to turn the cards over and to order them in a logical sequence of events. For each cartoon story sequenced correctly a maximum score of 6 can be obtained: two points are given each for the first and last correctly sequenced picture and one point each for the third and fourth picture. After the children correctly arrange the four cards, they are asked to answer questions that pertain to the mental states of the different characters, including questions of first-, second-, and third-order false beliefs as well as of reciprocity, deception and cheating detection. For each question answered correctly, one point is obtained. The variables of interest in this study were first-, second-, and third-order false belief (scores 0 to 3) and Total ToM (score 0–59).

#### Empathy

The Griffith Empathy Measure (GEM, Dadds et al., 2008) is a 23-item parent-report questionnaire adapted from Bryant’s Index of Empathy for Children and Adolescents (Bryant, 1982) in which parents rate the empathetic abilities of their child on a 9-point Likert scale from –4 (strongly disagree) to 4 (strongly agree). This questionnaire provides three scores: Cognitive empathy (score –56 to 56), Affective empathy (score –68 to 68), and Total empathy (score –92 to 92), with higher scores corresponding to higher levels of empathy. Cognitive empathy can be defined as “the ability to intellectually take the role or perspective of another person involving the ability to decode and label emotions and their situational cues” (Dadds et al., 2008, p. 112). Affective empathy is defined as “an affective response more appropriate to, or congruent with, someone else’s situation than to one’s own situation” (Dadds et al., 2008, p. 112). The GEM has adequate reliability and validity across gender and age (Dadds et al., 2008). In line with Decety and Cowell (2014), the Affective empathy score was used as the main measure of empathy, whereas the total and cognitive scores were used as complementary measures, in order to distinguish the specific link of each aspect of empathy with MR skills.

#### Affect Recognition

The Affect Recognition subtest from the NEPSY-II (Korkman et al., 2007) assesses children’s ability to recognize affect from pictures of children’s faces expressing one of five basic emotions (happiness, sadness, anger, fear, disgust) or a neutral expression. This 35-item task has four conditions increasing in difficulty (number of distractors; score 0 to 35). The total number of correct responses was used as the affect recognition variable in this study.

#### Moral Reasoning

**FIGURE 1:**
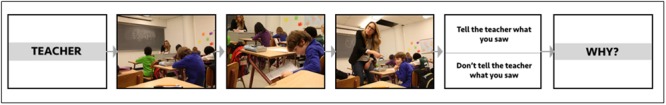
**Example item from the So-Moral-Child task (Socio-Moral Reasoning Aptitude Level)**.

The children’s version of the Socio-Moral Reasoning Aptitude Level task (So-Moral-Child, Dooley et al., 2010; Beauchamp et al., 2013) is a visual, computer-based task that presents nine moral dilemmas specifically designed for children and has gender-specific versions. Each dilemma (see example **Figure 1**) consists of an introductory screen presenting the name of the dilemma (e.g., teacher), three first-person perspective pictures of children actors playing out various social scenarios representing a conflict centered on the moral domain (e.g., concerns with justice, welfare/harm, and rights) according to Social Domain Theory (Turiel, 1983), and a final screen presenting a dichotomous decision (e.g., whether or not to engage in a particular action, such as stealing from a shop, cheating at a game, etc.). The aggregate number of responses is compiled to obtain a moral decision-making score, which ranges from 0 to 9 points. Children are then asked to provide a justification for their decision. Each participant’s justification is recorded verbatim and scored using a standardized coding system (Beauchamp and Dooley, 2012) based on the cognitive-developmental approach to MR (Kohlberg et al., 1983; Turiel, 1983; Gibbs, 2010). The moral decision-making score does not affect the moral justification score (both are independent). Developmental stages of MR have been adapted to fit the social nature of the dilemmas in the So-Moral task and consist of the following: (1) Centrations and authoritarian-based consequences; (2) Egocentric/pragmatic exchanges; (3) Interpersonal Focus; (4) Societal Regulation; and (5) Societal Evaluation (see **Table 1**). Transition stages (i.e., 1.5, 2.5, etc.) are used to account for answers that provide elements of two consecutive reasoning stages. When elements of non-consecutive stages are provided, the response is coded according to the highest schema detected. The MR maturity score (0–45 points) is obtained by summing the nine justification scores. This test has adequate inter-rater reliability and construct validity (Dooley et al., 2010). Each justification was scored independently by two trained raters (Intraclass correlation coefficient = 0.81) and discrepancies were resolved by discussion and consensus. The MR maturity score was used as the main dependent variable.

**Table 1:** Brief description of So-Moral coding and examples.

Level	Brief description	Example
**1**	Moral justifications have an egocentric focus, which is based on obedience to higher authorities and potential consequences of their actions for themselves (e.g., punishment). Thinking at this level is inflexible; there is only one right/wrong way to act.	• Because I could go to jail.
**2**	Moral justifications are based on a concept of pragmatic deals or exchanging favours with others (‘fair deals’). Thinking is more flexible and is determined by context. The correct option is the one that is right for oneself (self-interest).	• Because I might need his/her help in the future.
**3**	Moral justifications have a focus on interpersonal relationships, a sense of ‘good-ness’, and feelings such as empathy and trust. Decisions are made with good motives and a prosocial perspective of the world.	• Because he/she could get hurt.
**4**	Moral justifications start to incorporate a broader view of morality; based on the compliance with rules, regulations and standards that society has established to ensure social order.	• Because if everyone were to be unfaithful, relationships would not have any meaning.
**5**	Moral justifications are characterized by the capacity to evaluate situations from various points of view to identify values involved in the specific situation in order to make the fairest decision. Protection of fundamental values and people’s rights is specific to this stage, even though these concepts are expressed very concretely.	• Because people work hard for their things and we should respect their belongings.

#### Executive Functioning

Two tasks from the NEPSY-II battery (Korkman et al., 2007) and one task from the Wechsler Intelligence Scale for Children – Fourth Edition (WISC-IV^CDN-F^; Wechsler, 2005) were used to measure three executive functions (attentional control, inhibition, and verbal fluency).

##### Attentional control

The WISC-IV Cancellation subtest was used to measure children’s ability to direct their attention and resist distraction during a goal-oriented task. Children are asked to detect and mark objects belonging to the same category (i.e., animals) that are mixed among distracters, within a specific time limit. The raw total score of this task was used as the attentional control variable.

##### Inhibition

The NEPSY-II Inhibition subtest, an adaptation of the widely used Stroop task (Stroop, 1935/1992), assesses children’s inhibition skills by using images (shapes and arrows) instead of words. In the first condition (Naming), children look at a series of black and white shapes or arrows must name either the shapes (circles or squares) or the direction of arrows (up or down) as quickly as possible. In the second condition (Inhibition), children must look at the same series of black and white shapes or arrows, but must quickly name the opposite shapes or direction of arrow. In the third condition (shifting), children must alternate the response (same or opposite) depending on the color of the shape or arrow. The raw completion time score of the inhibition condition was used as the inhibition variable.

##### Verbal fluency

The NEPSY-II Word generation subtest was used to assess participants’ verbal fluency. In this task, children are asked to produce as many words as possible in 1 min, within a semantic and a phonetic condition. The raw semantic score was used as the verbal fluency variable.

### Statistical Analyses

Statistical analyses were performed using SPSS 21.0 software and the PROCESS macro for mediation and moderation analysis (Hayes, 2012, 2013). Prior to all statistical analyses, data were examined for any violations of test assumptions (normality, linearity, and homoscedasticity). Correlation coefficients were calculated to examine the relationship between MR maturity and age, socioeconomic variables (family income, parental education level, and a composite SES score including both variables), EF variables (attentional control, inhibition, and verbal fluency) and social cognition (ToM, empathy, and affect recognition). In order to examine the potential moderating and mediating effects of EF on the relationship between age and MR skills, we performed moderation analyses with each variable individually as potential moderators, and a parallel multiple mediation analysis with attentional control, inhibition and verbal fluency together as mediator variables, using the PROCESS macro provided by Hayes (2012). To assess the potential moderating and mediating effect of social cognition variables (affect recognition and third-order false beliefs, the ToM variable most correlated with MR) on the association between age and MR skills, we performed moderation analyses with each variable individually as a potential moderator, and a parallel multiple mediation analysis with both variables as a set of mediators. In mediation analyses, the statistical significance of the obtained regression coefficients was determined by obtaining a bias-corrected 95% confidence interval by bootstrapping based on 1,000 resamples. Finally, a three-step hierarchical multiple regression analysis was conducted with age in the first step, the EF most strongly associated with MR (i.e., verbal fluency) in the second step and social cognition variables that correlated with MR in the final step, in order to explore the specific contribution of demographic, cognitive, and socio-cognitive variables to MR maturity. Socioeconomic variables were not included in the models because they were not related to either MR maturity or other measures used in the study. Results corresponding to *p* < 0.05 were considered statistically significant. The strength of correlations and effect sizes were determined according to Cohen’s (1988) criteria.

## Results

### Descriptive Results

Participant demographic characteristics and participants’ results on socioeconomic, cognitive, and socio-cognitive measures are presented in **Tables 2** and **3**.

**Table 2:** Sociodemographic characteristics of the participants.

Variable	Frequency	*%*
***Gender***		
Male	36	52.6
Female	40	47.4
***Ethnic origin***		
White	72	94.7
Hispanic	2	2.6
African–American	2	2.6
***Total gross annual household income (CAN $)***		
Below 20 000 $	4	5.3
20 000 through 39 000 $	12	15.8
40 000 through 59 000 $	17	22.4
60 000 through 79 000 $	22	28.9
80 000 through 99 000 $	16	21.1
100 000 $ and more	5	6.6
***SES on the basis of the annual household income^1^***		
High SES	5	6.6
Middle SES	54	72.4
Low SES	16	21.1
***Maternal education***		
Master’s degree	1	1.3
Bachelor’s degree	10	13.2
College	29	38.2
High school graduate	28	36.8
Incomplete high school	8	10.5
***Paternal education***		
Doctoral degree	1	1.3
Master’s degree	2	2.6
Bachelor’s degree	4	5.3
College	15	19.7
High school graduate	35	46.1
Incomplete high school	16	21.1
Missing values	3	3.9

*^1^ Statistics Canada, 2015.*

**Table 3:** Main descriptive results and correlations of the study.

Variable	*M*	*SD*	Correlation		
			MR maturity	Age	SES composite
Age in months			0.54***	1	-0.02
***Socioeconomic variables***					
SES composite *z-*score	-0.03	0.75	-0.13	-0.02	1
Annual Household income *z-*score	0	1	-0.13	-0.05	0.73***
Maternal education *z-*score	0	1	-0.09	0.08	0.74**
Paternal education *z-*score	0	1	-0.04	-0.11	0.61***
***Cognitive variables***					
Intellectual functioning (WASI IQ)	108.6	9.9	0.17	0.16	0.03
Attentional control^a^	82.5	21.1	0.36**	0.64***	0.05
Verbal fluency^b^	30.3	9.3	0.49***	0.58***	-0.19
Inhibition^c^	74.9	26.0	-0.43***	-0.66***	
***Socio-cognitive variables***					
Affect recognition^d^	25.1	4.6	0.42***	0.54***	-0.22
Empathy (total score)^e^	33.2	23.4	0.21	0.34**	-0.09
Cognitive empathy^f^	25.5	14.2	0.22	0.32**	-0.10
Affective empathy^g^	23.8	18.8	0.19	0.29*	-0.07
Theory of Mind^h^	48.7	7.8	0.32**	0.44***	-0.08
First-order false beliefs^i^	2.7	0.6	0.11	0.27*	-0.05
Second-order false beliefs^j^	2.1	0.9	0.26*	0.40**	-0.08
Third-order false beliefs^k^	1.9	1.0	0.40***	0.36**	-0.03
Moral reasoning^l^	18.3	5.3		0.56	-0.13

*^∗^*p* < 0.05; ^∗∗^*p* < 0.01; ^∗∗∗^*p* < 0.001. ^a^WISC-IV Cancelation raw total score; ^b^NEPSY-II Word generation raw semantic score; ^c^NEPSY-II Inhibition raw completion time score; ^d^NEPSY-II Affect Recognition raw total score; ^e^Griffith Empathy Measure total score; ^f^Griffith Empathy Measure Cognitive empathy score; ^g^Griffith Empathy Measure Affective empathy score; ^h^Theory of Mind Picture Stories total score; ^i^Theory of Mind Picture Stories First-order false beliefs score; ^j^Theory of Mind Picture Stories task Second-order false beliefs score; ^k^Theory of Mind Picture Stories task Third-order false beliefs score; ^l^So-Moral, total justification score.*

### Association of Moral Reasoning Maturity with Socioeconomic Factors

Moral reasoning maturity was not related to socioeconomic variables including family income and maternal and paternal education level (see **Table 3**).

### Association of Moral Reasoning Maturity with Age, Executive Functioning, and Social Cognition

As shown in **Table 3**, MR maturity was strongly and directly associated with age (*r* = 0.54 *p* < 0.001). In terms of EF, there was a moderate significant relationship between MR maturity and inhibition, as well as verbal fluency, and a small but significant relationship between MR and attentional control. Concerning social cognition variables, MR maturity was moderately associated with total ToM and higher order ToM, as well as with affect recognition, but it was not associated with first-order false beliefs. Affective empathy, our main empathy variable, as well as total empathy, were not associated with MR skills, but cognitive empathy approached significance (*r* = 0.22 *p* = 0.054).

### Age and Moral Reasoning Maturity: The Moderating and Mediating Role of EF

Results of the moderation analyses performed using the PROCESS macro detected no significant interactions of age X attentional control, (*b* = –0.0009, 95% CI [–0.0016 –0.0033], *t* = 0.7155, *p* = 0.48); inhibition (*b* = –0.0001, 95% CI [–0.0031 –0.0029], *t* = –0.0671, *p* = 0.95) and verbal fluency (*b* = –0.0014, 95% CI [–0.0068 –0.0040], *t* = –0.5167, *p* = 0.61) on MR maturity, indicating that these EF do not play a significant moderating role in the relationship between age and MR maturity.

**Table 4** displays the bootstrapped estimates for the total and specific indirect effects obtained from multiple mediation analysis. The total indirect effect of age on MR skills through EF variables as a group was not statistically significant, as the 95% confidence intervals contained zero. Considering the possibility of having significant specific indirect effects in the absence of a significant total indirect effect (Preacher and Hayes, 2008), we evaluated the specific indirect effects associated with each of the three mediators, detecting a significant mediating role for verbal fluency on the link between age and MR skills. To assess the effect size of verbal fluency as a mediator, we performed a simple mediator model. The indirect effect of verbal fluency obtained was statistically significant (*b* = 0.041, 95% CI [0.0013 0.0890]), as the confidence intervals do not contain zero. The Kelley–Kappa squared obtained (κ^2^; Hayes, 2013) indicates an effect size of 0.15, which is considered a medium effect according to Preacher and Kelley (2011), based on Cohen’s (1988) criteria.

**Table 4:** Indirect effects of age on MR skills via EF and social cognition.

Mediator	Bootstrap estimate	SE	BC 95% CI lower	BC 95% CI upper
***Model 1 EF***				
Attention control	–0.0079	0.0212	–0.0498	0.0352
Inhibition	0.0148	0.0250	–0.0336	0.0638
Verbal fluency	0.0336	0.0216	0.0030	0.0887
Total indirect effect	0.0465	0.0299	–0.0148	0.1075
***Model 2 social cognition***				
Affect recognition	0.0272	0.0146	–0.0043	0.0560
Third-order false beliefs	0.0223	0.0131	0.0044	0.0574
Total indirect effect	0.0485	0.0179	0.0195	0.0923

*Based on 1,000 bootstrap samples. BC, bias corrected; CI, confidence interval.*

### Age and Moral Reasoning Maturity: The Moderating and Mediating Role of Social Cognition

Results of the moderation analyses also detected non-significant interactions of age X Third-order false beliefs (*b* = –0.0483, 95% CI [–0.1080 –0.0114], *t* = –1.6131, *p* = 0.11) and affect recognition (*b* = –0.0011, 95% CI [–0.0089 –0.0111], *t* = 0.2141, *p* = 0.83) on MR maturity, indicating that social cognition variables do not moderate the relationship between age and MR maturity. **Table 4** displays the bootstrapped estimates for the total and specific indirect effects obtained from multiple mediation analysis. The total indirect effect of age on MR skills through third-order false beliefs and affect recognition as a set was statistically significant, as the confidence intervals do not contain zero. The specific indirect effect of each variable was evaluated, detecting a significant mediating role of third-order false beliefs on the link between age and MR skills. The effect size of third-order beliefs as mediators was also assessed performing a simple mediator model. The results indicate a significant indirect effect (*b* = 0.0224, 95% CI [0.0041 –0.0571]) with a medium effect size (κ^2^ = 0.09).

### Contribution of Demographic, EF, and Socio-Cognitive Variables to Moral Reasoning Maturity

As shown in **Table 5**, a three-level hierarchical multiple regression indicates that in Step 1, age contributed significantly to the regression model, *F*(1,73) = 28.129, *p* < 0.001 and accounted for 28% of the variation in MR maturity, with a large effect size (*f*^2^= 0.37). Introducing verbal fluency to the model explained an additional 5% of variation in MR scores and the change in *R*^2^ was significant, *F* change (1,72) = 5.571, *p* = 0.021. Together, age and verbal fluency explained 33% of MR scores (*F*(2,72) = 17.731, *p* < 0.001), which corresponds to a large effect (*f*^2^= 0.49). Finally, the addition of social cognition variables (third-order false beliefs and affect recognition) to the regression model explained an additional 6% of the variation in MR maturity and the change in *R*^2^ was also significant, *F*(2,70) = 3.426, *p* = 0.038. Together, age, verbal fluency, third-order false beliefs and affect recognition explained 39% of MR scores [*F*(4,70) = 11.176, *p* < 0.001], which corresponds to a large effect (*f*^2^= 0.64). When social cognition variables were included in step 3 of the regression model, verbal fluency (β = 0.24, 0.041) and third-order false beliefs (β = 0.22, *p* = 0.030) were significant, independent predictors of MR maturity.

**Table 5:** Contribution of demographic variables, EF, and social cognition to moral reasoning maturity.

Predictor	MR Maturity	
	Δ*R*^2^	β
***Step 1***	0.28^∗∗∗^	
Age		0.53^∗∗∗^
***Step 2***	0.05^∗^	
Age		0.37^∗^
Verbal fluency		0.28^∗^
***Step 3***	0.06^∗^	
Age		0.23
Verbal fluency		0.24^∗^
Affect recognition		0.16
Third-order false beliefs		0.22^∗^
***Total *R*^2^***	0.39^∗^	
*n*	76	

*^∗^*p* < 0.05; ^∗∗∗^*p* < 0.001.*

## Discussion

This study explored the relationship between demographic factors, EF, social cognition, and children’s moral maturity, as well as the independent and combined contribution of these variables to MR.

While several studies have found a link between demographic factors, such as maternal education and SES, and moral processes (Dunn et al., 2000; Arsenio and Gold, 2006; Hinnant et al., 2013), others have not reported such an association (Caravita et al., 2012; Vera-Estay et al., 2014). In the current study, neither the SES composite *z*-score, nor parental education and income individually were associated with children’ MR skills. This discrepancy in results may be due to population differences and the relatively high-level of SES in the current sample and absence of any real social risk. Despite adequate variability in family income in our sample, 98% of the mothers of participants had at least some high school education, possibly off-setting the adverse effects of low SES, and contrasting with studies in extremely disadvantaged children (e.g., Arsenio and Gold, 2006). In addition, methodological differences could contribute to explaining the lack of association between SES and MR in this study. Parental education is known to be linked to child academic competence, including reading and writing skills (Bradley and Corwyn, 2002; Janus and Duku, 2007; Hooper et al., 2010), yet the So-Moral task was specifically designed to remove the need for reading and writing for successful performance (Dooley et al., 2010), therefore potentially eliminating the association between parental education and MR. Further studies with a larger sample and greater SES and social risk variability may be necessary to clarify the exact role of parental education and SES on MR.

On the other hand, consistent with cognitive-developmental theories (Gibbs, 2013) and recent studies combining neurophysiological and behavioral measures (Decety et al., 2012), the current results confirm the positive association between age and MR skills suggesting an evolution of these during the transitional period from childhood to pre-adolescence, in line with the results obtained in our study with early to late adolescents using the So-Moral task (Vera-Estay et al., 2014).

This developmental tendency may be explained by differences in biological and cognitive maturity, but also by increased social experience and opportunities for social perspective taking. During the school years, children participate actively in the construction of social relationships and learn to adjust their behavior in order to be integrated in the community, especially in peer groups. With age, children learn to integrate and coordinate social and moral knowledge (Killen and Rutland, 2011), but this progressive process is influenced by the control of egocentric bias in order to consider other’s perspectives (Gibbs, 2013). In our study, younger children tended to justify moral decisions on the basis of egocentric and concrete reasons such as obeying authority (e.g., parents, teacher), avoiding punishment, and obtaining direct personal benefits, whereas older children more frequently incorporated a socio-centric focus in their reasoning, including aspects such as empathy toward the victim, caring and maintaining interpersonal relationship, and more abstract concepts such as mutual respect, reciprocity, and responsibility.

Despite the age-related tendency of MR skills in typically developing children, age alone was not sufficient to explain individual differences in MR. We therefore explored associations with other cognitive and socio-emotional factors in order to shed light on the building blocks of MR.

### Moral Reasoning and Executive Functioning

Concerning the cognitive factors related to MR skills, three aspects of EF (attentional control, inhibition, and verbal fluency) had a small to moderate association with MR skills, with verbal fluency having the strongest association. Globally, these results are in line with previous research investigating the contribution of EF to social adaptation (Kochanska et al., 2000; Eisenberg et al., 2004; Martel et al., 2007; Beauchamp and Anderson, 2010; Miller and Hinshaw, 2010) and moral understanding (Decety and Howard, 2013), as well as with our previous study which found a positive link between MR and several EF (conceptual reasoning, cognitive flexibility, verbal fluency, and feedback utilization), in addition to the independent contribution of cognitive flexibility and verbal fluency to MR maturity (Vera-Estay et al., 2014).

The relationship between attentional control and MR skills highlights the importance of directing, focusing and maintaining attention on relevant stimuli during social situations. Whereas paying attention to social clues is considered the first step in social understanding according to Crick and Dodge’s (1994) Social Information Processing Model, this result suggests that this skill is also specifically related to better MR. Thus, detecting the relevant information contained in the So-Moral task (as in everyday life) requires individuals to focus on the story, control environmental, and internal distractors, and pay attention to subtle visual details, in order to accurately interpret the situation that will then be evaluated according to moral processes.

Inhibition skills were also related to more mature MR. This result is consistent with Barkley’s (1997) established ADHD model, which depicts inhibition as a pivotal skill for adequate self-regulation and social adaptation, one which sustains high order socio-cognitive processes, such as social perspective taking, internalization of speech and MR. In the same vein, Birch and Bloom (2004) have underscored the role of inhibition in cognitive perspective taking, specifically false belief understanding, since it requires the capacity to control and suspend automatized subjective points of view, in order to think about other’s mental states and knowledge. During MR, inhibiting one’s own point of view in favor of taking another’s perspective could enable people to distinguish between intentional and involuntary harm, or suspend the analysis of personal benefits/costs in a moral decision in order to evaluate the potential effects of these decisions on other people. Besides, inhibition skills work together with attentional control during the encoding step of the Social Information Processing Model, helping to control for the influence of distractive or irrelevant stimuli and stop disruptive behaviors, in order to focus on significant social and moral cues. Of note, the finding of a significant association between attentional/inhibitory skills and moral maturity suggests that future work should investigate the quality of MR in children presenting deficits in these areas, such as those with ADHD.

The influence of inhibition on internalization of speech, verbal fluency, and MR proposed by Barkley (1997) is also consistent with our finding concerning the positive link between verbal fluency and MR skills. Indeed, the ability to rapidly access vocabulary may favor inner speech fluency and consequently the MR process, considered as an inner moral dialog in which social situations are evaluated in light of moral criteria (Tappan, 1997). Discussions of moral issues with peers (Berkowitz and Gibbs, 1983; Walker et al., 2000) and parents (Walker and Taylor, 1991; Walker et al., 2000) have been shown to be pivotal in MR development, further underscoring the importance of fluid verbal skills. Therefore, besides the potential influence of verbal fluency on the internal MR process, this EF may also support the exercise of MR skills in social contexts fostering active participation in moral dialogs and debate with others.

### Moral Reasoning and Socio-Cognitive Skills

This study also investigated the relationship between MR and three important socio-cognitive skills in maturation during childhood: affect recognition, empathy, and ToM.

Facial emotional expressions are powerful clues for an adequate understanding of moral situations, since they inform the internal affective state of the actors involved. However, traditional written MR tasks do not offer this basic source of information. Taking to account this limitation, our study measured MR with a visual task including pictures or real child actors in which facial emotional expressions reflect the moral situation played out, and included an affect recognition task developed for the pediatric population. Using this approach, we found that children who are better at recognizing emotions had more mature levels of MR, confirming the importance of emotional information for a deeper comprehension of moral issues. This result is consistent with previous research relating deficits in facial affect recognition with psychopathy in youth and adult offenders (Hastings et al., 2008; Bowen et al., 2014), as well as in children with psychopathic tendencies (Blair et al., 2001a; Stevens et al., 2001). Along with the association of MR with both attentional control and inhibition skills, the link between affect recognition and MR skills is a novel and interesting finding that highlights the role of encoding cues on high-order social cognition, namely, MR skills. While attentional control and inhibition support the encoding process, facial emotional expressions play the role of content or relevant clue to be encoded. The aptitude to distinguish these emotions fosters the second step of social information processing (Crick and Dodge, 1994), i.e., the interpretation of the encoded cues, providing rich information to infer more complex mental states to the actors involved in a given social situation (e.g., he is sad to have been rejected by a friend).

Contrary to our hypothesis, children’s affective empathy, as reported by their parents, was not related to their MR skills. While unexpected, this result is, however, in keeping with Decety and Cowell (2015)’s claims that empathy does not always lead to moral behavior and could sometimes affect the objectivity of MR. In children, affective empathy may, for instance, push them to protect a child from being bullied, but may also induce in-group bias justifying immoral behaviors in order to protect or help friends, interfering in the analysis of the consequences on other actors in the situation. The impact of in-group bias in children’s moral evaluations has also been demonstrated in a recent study of children aged 3–9 years, who considered within-group harm as wrong regardless of explicit rules, but between-group harm as wrong when explicit rules are present (Rhodes and Chalik, 2013). Thus, mature MR skills may help children to analyze the situation and expand their concern beyond spontaneous and evolutionarily older mechanisms such as kin-care and in-group bias, to humanity as a whole. Perspective-taking skills have a key role in reducing group partiality and upholding the global application of moral values (Decety and Cowell, 2015).

In line with this view, our study also found that ToM skills, in general, as well as higher-order levels of false belief understanding are associated with better MR skills. However, whereas basic understanding of other’s minds (first-order false beliefs) was not associated with MR, more complex understanding represented by second-order and third-order false beliefs (e.g., “What does the boy expect the girl thinks he wants to do?”) was related to better MR skills in childhood. Third-order false beliefs support theoretical reasoning about intentions, lies, cheating, deception, and so on. Hence, along with affect recognition, third-order false belief theoretically contribute to MR processing in the second step of social information processing (Crick and Dodge, 1994), sustaining the interpretation of cues in order to evaluate the moral situation, in addition to being the cognitive basis of social perspective taking.

### Age and Moral Reasoning Maturity: The Moderating and Mediating Role of EF and Social Cognition

As described above, while the association between age and MR maturity has been documented theoretically and empirically (Decety et al., 2012; Gibbs, 2013), the multiple factors influencing this developmental trend continue to be explored. The second and third aims of this study were to investigate to what extent the link between age and MR maturity was moderated or mediated by EF and social cognition. According to our results and contrary to our hypothesis, neither EF nor social cognition variables moderated the effect of age on MR maturity. This result suggests that, in healthy children, variations in EF and social cognition do not affect the magnitude of the relationship between age and MR skills. Further research including children with EF and/or social cognition deficits are necessary to determine the generalizability of the results. On the other hand, verbal fluency and third-order false beliefs had a moderating role in this link, in line with cognitive-developmental views of MR, suggesting that the development of specific thinking processes related to internal speech and perspective taking help to explain the increase in moral maturity with age.

In light of growing societal concern regarding the consequences of children’s behaviors, such as bullying and peer exclusion, for both victims and victimizers, it is necessary to create effective strategies to foster appropriate MR skills at an early age. Understanding the mediators of the effect of age on MR contributes to this challenge and provides a starting point for developing targeted intervention programs for the improvement of social skills in both typically developing and clinical populations. The results obtained here provide direction for such programs, particularly considering that both verbal fluency and ToM have the potential to be targeted in socio-cognitive intervention programs.

### Combined Contributions of Demographic, Executive Functioning, and Social Cognition to Moral Reasoning

The fourth aim of this study was to investigate the relative and combined contributions of age, EF, and socio-cognitive variables to children’s MR skills. To enhance power, the three-step hierarchical regression conducted included the variables most correlated with MR skills (i.e., age, verbal fluency, third-order false beliefs, and affect recognition).

Together these factors explained 39% of the variation in children’s MR skills, each domain making an independent contribution. As expected in a sample of healthy children, the contribution of age (introduced in first step) had a large effect on MR scores, supporting the cognitive-developmental approach of morality (Gibbs, 2013) and in line with studies demonstrating that developmental disorders may interfere with this age trend (Anderson et al., 1999; Blair et al., 2001b; Couper et al., 2002; Dooley et al., 2010; Beauchamp et al., 2013). The addition of verbal fluency (added in the second step) had a small but significant independent contribution to MR scores, as well as third-order false beliefs and affect recognition (added in the third step). In the final model, children’s verbal fluency and their ability to understand third-order false beliefs were independent predictors of MR maturity. The current study indicates that these two skills, theoretically at the core of MR, are not only associated with MR skills, but also serve as cognitive predictors in healthy children, suggesting that subtle variations in these skills may have an impact on the MR process. Considering that EF and social cognition are impaired in several developmental disorders of childhood affecting social understanding, such as autism spectrum disorder and attention deficit hyperactivity disorder (Korkmaz, 2011), this study provides strong support for investigating MR processes in such groups.

### Strengths and Limitations

This study provides novel information about the contribution of socio-cognitive building blocks of children’s MR skills, on the basis of the results of an ecological approach to the measurement of MR for pediatric populations. Our sample has an adequate balance concerning the sex and age of the participants, which enhances generalization of the results. Moreover, the results obtained with healthy children shed light on potential loci of intervention for children at-risk for socially maladaptive behaviors. However, the present study has some limitations that need to be taken into account. First, MR is a higher-order human skill influenced by multiple interactive factors. Many of these were taken into account here in one of the few studies to explore their combined contribution; however, a modest sample size limited inclusion of additional environmental, cultural, and individual variables (including other EF) that could also contribute to MR. These could be explored in a larger sample, with more variability in terms of maternal education, SES and ethnicity, in order to build an even more comprehensive view of moral development. Although a strength of the study was that it relied on direct neuropsychological measures, empathy was assessed using a parent-report questionnaire, which may have introduced bias. Direct observations of children’ empathetic skills/behaviors could better estimate their relationship with MR. Finally, whereas our study observed age differences in MR skills in a cross-sectional design, further studies using a longitudinal design could more accurately explore the progression of MR during childhood and infer causal relationships between the variables explored.

## Author Contributions

All authors contributed to study conception and design. EV-E and MB contributed to the development of the study hypotheses. Testing and data collection were performed by EV-E, AS, and CC. EV performed the data analysis and interpretation under the supervision of MB. EV-E drafted the manuscript with input from MB. MB, AS, and CC provided critical revisions. All authors read and approved the final version of the manuscript for submission.

## Conflict of Interest Statement

The authors declare that the research was conducted in the absence of any commercial or financial relationships that could be construed as a potential conflict of interest.
